# Book Review: La Aventura Praxiológica. Ciencia, Acción y Educación Física

**DOI:** 10.3389/fpsyg.2020.02084

**Published:** 2020-08-28

**Authors:** Maria Pilar Founaud, Asier Oiarbide

**Affiliations:** ^1^Department of Didactics of the Musical, Plastic and Corporal Expression, Faculty of Education, University of Zaragoza, Zaragoza, Spain; ^2^Department of Physical Education, Faculty of Education and Sports, University of the Basque Country, Vitoria-Gasteiz, Spain

**Keywords:** physical education, motor praxeology, games, sports, transdisciplinarity

In 1959 Pierre Parlebas, just graduated from the Normal Higher School of Physical Education (ENSEP) in Paris, published his first, visionary article in response to an invitation from one of his former teachers: É*ducation physique et éducation philosophique* (Parlebas, 1959). In 1986 he successfully applied for the position of Professor in the Faculty of Social and Human Sciences of La Sorbonne, after receiving a State Doctorate in the same university, 2 years earlier, thanks to a piece of research in three volumes that put traditional sporting games in the front line of physical education and social sciences: *Psychologie sociale et théorie des jeux: étude de certains jeux sportifs* (Parlebas, 1985). In between these two biographical milestones, Parlebas built up an outstanding theory of human motricity, games and sports, and physical education whose inception and development are now fully accessible to Spanish readers thanks to this work*: La aventura praxiológica. Ciencia, acción y educación f*í*sica* (The praxeological challenge. Science, action, and physical education).

This volume of 496 pages, published thanks to the support of The Andalusian Regional Government, contains 32 articles selected and translated by the editor Raúl Martínez-Santos (Martínez-Santos, 2017), who groups them chronologically and thematically in seven parts. As he explains in a sound introduction in which Parlebas' thought and trajectory are wisely exposed and explored: “There is a chance that Parlebas be more famous that truly known, despite his sports classification is most popular and many of his concepts (i.e., motor conduct, motor situation, internal logic, sports games, etc.) already make part of the terminology of so many scholars and practitioners” (Parlebas, 2017). We are sure that this book will change this situation for the better because the selection and translation of the references result in an instructive, meaningful whole that increases the individual value of each chapter. Besides, the numerous translator's and editor's notes help readers of any level contextualise and understand the whys and wherefores of Parlebas' proposals. Every part is opened with a brief introduction by Parlebas. Along with the presentation of the book and the second chapter (titled *The drunken boat* after Rimbaud's poem, Parlebas, 2014), these short recollections compose a little treasure with great academic value for the opportunity they give the readers to share Parlebas' reflections on his own work. In this sense, the opening part includes the two aforementioned articles as kind of the alpha and omega of a never-ending alphabet.

*Revue Éducation Physique* & *Sportif*, the journal founded in 1950 by the association of alumni of the ENSEP, was the main venue for Parlebas to present his scientific, academic production. In 1990, the third edition of *Dossiers EP*&*S n*° *4: Activités physiques et éducation motrice* contained the fundamental corpus of his literature, and the core of this dossier can be found in this volume. The second part of the book contains his introducing, position taking article: “A physical education in shreds” (Parlebas, 1967). In this long article split up in four numbers of the journal Parlebas presented a thesis surprisingly contemporary: physical education suffers from a double shredding that involves a multiplicity of intervention domains, on the one hand, and a plurality of doctrines and methods, on the other hand. This situation is severely weakening and endangers the legitimate ambition of the whole profession, which suffers from a sempiternal serfdom. The way-out from the crisis depends on the recognition of the “motor conduct,” the meaningful organization of motor behaviors, as the common factor of games, sports, and any physical activity, and, consequently, as the proper object of object physical education. In this article, “sociomotricity” is also presented as operationally distinct than a redefined ‘psychomotricity.”

Parlebas had the chance to enjoy the effervescence of the French social sciences in the 1960s. His studies in psychology, sociology, linguistics, and mathematics in La Sorbonne allowed him to develop a *structural, not structuralist* theory of motor action that, given time, would be proposed as a theory of motor action, or *motor praxeology*. The third part contains seven articles, probably the densest ones, on motor learning and transfer, gestalt theory, sociometry, genetic epistemology, and the cognitive and affective conditions of motor action that would lead to an innovative classification of sporting games with eight different action domains regarding the relationships that agents establish with their social and natural milieus. The theory of motor action will be completed in the 1970s with the mathematization of the internal logic of sporting games with the so called “ludomotor universals.” The presentation of these models, including the two ones related to the game-changer “semiotricity” (semiology of the motor action), is the content of the fifth part, in which traditional games like “sitting ball” and “fresher” are the most appropriate examples.

The other three parts are more diverse, equally important, and certainly more surprising. One of the great values of this publication is to complete Parlebas' bibliography in Spanish with his most physical-educational papers. Parlebas is mainly known as a sociologist (Parlebas, 1988) and a theoretician (Parlebas, 2001), but he is first and for all a teacher of physical education whose great experience in the field takes himself to question the foundations of his art and science. The fourth part is all about physical education's elements: contents like body expression and dance, higher education of teachers, institutional value and consideration, benefits and outcomes, games, dreams, and fantasies… In this vein, the sixth part contains four short texts in which the alleged superior value of sports practice is contested in a different style: these texts were written for practitioners off college, sports and summer camps monitors willing to explore outside the bounds of competition, what make them even more valuable. Parlebas learnt about the potential of traditional games during sleep-away sporting camps, in which he could put to the test, in a masterful way, the received theories about teaching methods and alleged superiorities.

The seventh, closing part includes four articles that present Parlebas' ideas in their maturity. These later works on motor metacommunication, sports, and epistemology of physical education, are a treatise on the three elements that give sense to a whole life dedicated to physical education: the deep respect for the right to be individually considered in education, the continuous research for a better understanding of culture and society, and the total commitment to the scientific foundations and independence of a wonderful profession. *La aventura praxiológica. Ciencia, acción y educación f*í*sica*, is a long waited work that comes to be a fundamental contribution in the development of motor praxeology, physical education, and traditional games.

## Author's Note

*La aventura praxiológica. Ciencia, acción y educación f*í*sica* is the adventure that Pierre Parlebas undertakes to create the science of motor action, a new conception of physical education, the process of creation of a matrix science, a change of paradigm for an orphan, as physical education has been. It is an essential book for any scientist, teacher or agent connected to physical sporting activities. It can be considered a prequel of É*lémentes de sociologie du sport* (Parlebas, 1988) and *Jeux, sport et société: lexique de praxéologie motrice* (Parlebas, 2001). After its reading, these two later publications in his biography take on a fuller meaning. This book is an essential reference for all scholars of the sciences of physical activity and sport that shows the scientific biography of a brilliant and unusual man who has created and placed praxeology among the other sciences, showing the relevance and self-same material it contains: the motor action.

## Author Contributions

All authors listed have made a substantial, direct and intellectual contribution to the work, and approved it for publication.

## Conflict of Interest

The authors declare that the research was conducted in the absence of any commercial or financial relationships that could be construed as a potential conflict of interest.
